# An Accessible Web-Based Survey to Monitor the Mental Health of People With Mild Intellectual Disability or Low Literacy Skills During the COVID-19 Pandemic: Comparative Data Analysis

**DOI:** 10.2196/44827

**Published:** 2024-05-30

**Authors:** Monique CJ Koks-Leensen, Anouk Menko, Fieke Raaijmakers, Gerdine AJ Fransen-Kuppens, Kirsten E Bevelander

**Affiliations:** 1 Department of Primary and Community Care Radboud university medical center Nijmegen Netherlands; 2 Academic Collaborative Intellectual Disability and Health - Sterker op Eigen Benen (SOEB) Nijmegen Netherlands; 3 Academic Collaborative AMPHI - Integrated Health Policy Nijmegen Netherlands; 4 Safety and Health Region Gelderland-Midden Arnhem Netherlands; 5 Municipal Health Service Gelderland Zuid Nijmegen Netherlands

**Keywords:** monitoring, mental health, intellectual disabilities, low literacy, COVID-19, web-based survey

## Abstract

**Background:**

The COVID-19 pandemic and related control measures affected the mental health of all populations. Particular subgroups are underrepresented in mainstream surveys because they are hard to reach, and study measurements are not adapted to their skills. These subgroups include people with lower cognitive and literacy skills, such as people with mild intellectual disability (MID), who were considered vulnerable during the COVID-19 pandemic given their low socioeconomic status, small social networks, increased risks of health problems, and difficulties understanding health-related information.

**Objective:**

This study examines the impact of the COVID-19 pandemic on mental health among people with MID or low literacy skills compared with those predominantly represented in national surveys.

**Methods:**

A repeated cross-sectional study of people with MID or low literacy skills and a general population sample was conducted in the Netherlands. An easy-read web-based survey was co-designed with, and tested among, people with MID or low literacy skills and conducted in 3 rounds within 1 year of the COVID-19 pandemic (T1: November to December 2020, T2: March to April 2021, and T3: September to October 2021). The survey contained questions about demographics and 6 aspects of mental health: feeling happy, feeling energized, feeling stressed, worry, feeling lonely, and sleeping problems.

**Results:**

Our adapted survey and recruitment procedure enabled 1059 persons with MID or low literacy skills to participate (T1: n=412, 38.9%; T2: n=351, 33.1%; and T3: n=296, 28%). They were significantly younger, had a lower level of education, and more often than not were born outside the Netherlands compared to the general population sample (*P*<.001). Approximately half of them (604/1059, 57.03%) received professional care. They displayed poorer mental health scores than the general population sample. The percentages of people with MID or low literacy skills who reported more negative feelings in T1 ranged from 20.6% (85/412) reporting feeling lonely *often* or *almost always* to 57.8% (238/412) reporting feeling happy *almost never* or *sometimes*. The general population sample’s percentages were 5.4% (160/2930) and 32.2% (941/2918), respectively. Although scores improved over time in both populations, the disproportional effects remained.

**Conclusions:**

General COVID-19–related restrictions for the entire Dutch population affected people with MID or low literacy skills more negatively than the general population. Our study underscores the relevance of including these subpopulations in public health research because they are often overlooked in regular health data. An accessible web-based survey particularly targeted at this population enabled us to do so, and we reached a group of respondents significantly different from regular survey participants. This survey’s results provided insights into the health of people with MID or low literacy skills and gained knowledge to be used by care organizations and policy makers to reduce health disparities during a pandemic and in general.

## Introduction

### Studying COVID-19–Related Impact

The COVID-19 pandemic and related disease control measures affected the entire world. People were advised to adhere to strict hygiene measures and to work from home (if possible), and public places and nonessential shops were closed. In addition, social distancing and visiting restrictions were in place during lockdowns. In general, these restrictions had a major impact on daily routines, social contacts, and mental health [1-3], affecting some individuals more than others [4]. In the Netherlands, the *Rijksinstituut voor Volksgezondheid en Milieu* (RIVM; National Institute for Public Health and the Environment) started conducting a national longitudinal survey to monitor the impact of COVID-19 and related measures on Dutch citizens [5,6]. The monitor is informative regarding disease control strategies and policy making [7]; however, there is an external validity bias because participant characteristics show that a majority of the participants have a high educational level and are middle-aged or older and women [6,8,9]. Indeed, studies have demonstrated that particular subgroups are often underrepresented and excluded from mainstream surveys because general recruitment strategies are unsuitable for reaching them, and study measurements are not adapted to their cognitive level or literacy skills [10-12]. These groups often comprise people with lower socioeconomic status and higher risks of health problems, and, in the case of the COVID-19 pandemic, more difficulties complying with preventive measures [13-15], given their housing or work situation.

This study examines the impact of the COVID-19 pandemic and related restrictions on mental health and well-being among people with lower cognitive and literacy skills in addition to those who are predominantly represented in the national survey. An accessible survey based on the national survey was developed, and alternative recruitment techniques were used to specifically include these underrepresented subgroups.

### Subpopulations at Risk for Greater COVID-19–Related Impact

In the Netherlands, approximately 19% of the adult population (ie, 2.5 million adults) have limited reading, writing, or numeracy skills [16]. These limitations have various causes, such as a low level of educational attainment, migrant background, parents’ level of education and literacy, or low information-processing skills [17]. The last item plays an important role in people with mild intellectual disability (MID), who experience considerable limitations in both intellectual functioning and adaptive behavior and often need support in their daily life [18]. It is estimated that 4% to 8% of the Dutch population have an MID [19]. People with low literacy skills or MID often have limited work and income, poor health, and small social networks [20-24]. In general, studies have shown that people with low education and health literacy as well as those without social support, a stable income, a daily routine, and access to services are more at risk of mental health problems such as anxiety, general distress, and loneliness arising from the COVID-19 pandemic [1,3,5,14,25,26]. Therefore, it is likely that the COVID-19 pandemic had a higher impact on the mental health of people with MID or low literacy skills compared with the general population. However, during the first months of the pandemic, very limited knowledge was available about the impact on this subpopulation, and our study was set up to provide both these essential insights and practical recommendations for policy makers and care providers.

During the COVID-19 pandemic, studies on mental health specifically aimed at people with mild or more severe intellectual disability (ID) showed negative impacts as a result of social isolation or a lack of social support, the rapid changes in COVID-19–related measures and difficulty understanding these measures, difficulty accessing services, and disruption of daily routines [27-29]. Two European surveys among people with ID found that more than half reported stress or anxiety [30,31] or felt more anxious than usual because of the pandemic and subsequent lockdown [31]. A US survey found that 41% of the participants with ID had experienced more mental health problems or symptoms since the pandemic began; worry and stress were most often mentioned [32]. Similarly, people with low health literacy experienced more anxiety disorders, bouts of depression, and sleeping disorders during the COVID-19 pandemic than those showing sufficient health literacy [14]. Altogether, these studies—primarily conducted during the first lockdown periods—showed a great impact on the mental health of people with MID or low literacy skills, which contributed to an increase in preexisting inequalities in health and well-being [24,33].

The current underrepresentation in national surveillance and surveys of people with MID or low literacy skills, as well as the consequent lack of information about them, adds to existing health disparities. To better understand the impact of the COVID-19 pandemic on people with MID or low literacy skills, the specific factors driving this impact, and their specific needs, more information is urgently needed. Knowledge acquired through monitoring population health in its local context can provide a basis for government and health organizations to develop appropriate strategies to reduce this impact accordingly. In addition, the course of the pandemic and the ever-changing COVID-19-disease control strategies over time are important aspects regarding the context in which people were affected. Due to the rapidly changing situation and regulations during the pandemic (eg, when vaccinations were offered or restrictions were lifted), a dynamic impact on mental health was expected, and more insight is needed into how people responded to this unpredictable course.

### Objectives

This study examines the impact of the COVID-19 pandemic on people with MID or low literacy skills in direct comparison with the general population and over the course of the pandemic at 3 different time points. A unique survey study was set up that complemented the RIVM national survey. This study developed an accessible version of the web-based survey together with representatives of the target population and used suitable techniques to reach people with MID or low literacy skills.

## Methods

### Study Design

A repeated cross-sectional study of people with MID or low literacy skills and a general population sample was conducted during the COVID-19 pandemic in the Netherlands. The inclusion criteria were people with reading and writing difficulties, aged ≥16 years, living in the Netherlands, and completion of the survey. No exclusion criteria were used. A control question to assess participants on literacy skills or intellectual ability was not included because this was expected to be too sensitive for the participants. For reasons of comparison, the same survey was presented to 2 panels used to represent the general Dutch population.

**Figure 1:**
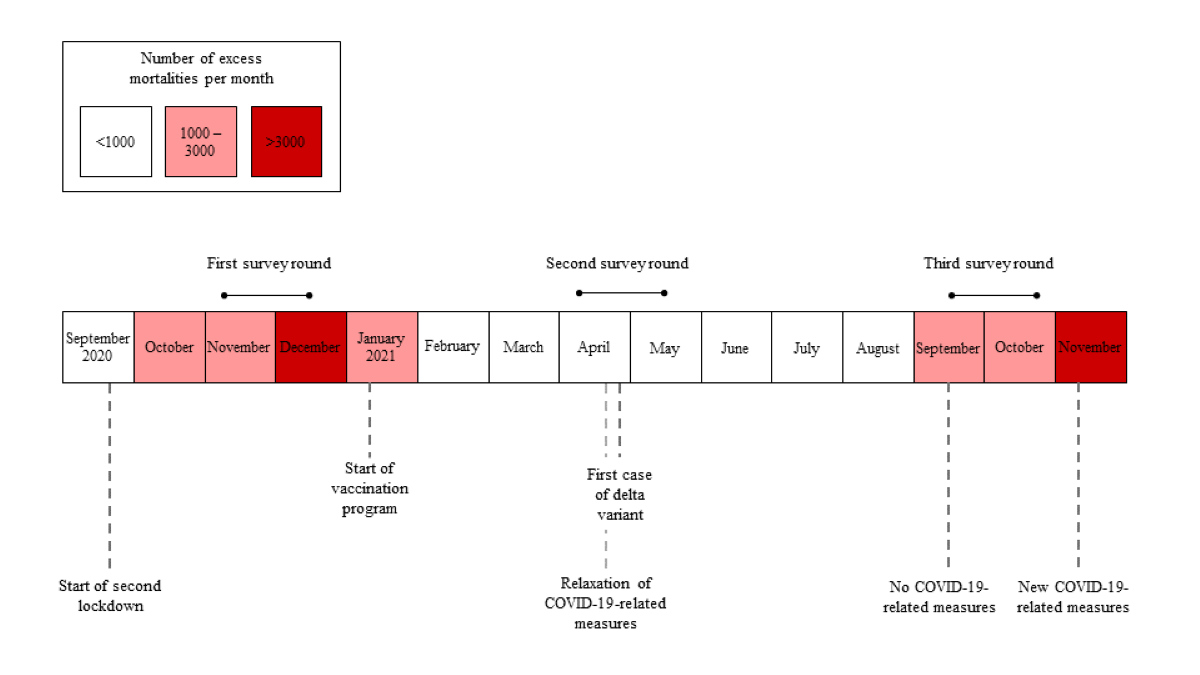
Timeline showing COVID-19–related measures, COVID-19 mortality cases, and the timing of survey periods. The number of excess mortalities indicates the severity of the COVID-19 waves.

The survey was administered 3 times in a 1-year period between November 2020 and November 2021. The first survey (T1) was distributed during a nationwide second lockdown (November to December 2020), the second survey (T2) was administered after the second lockdown and when the Dutch vaccination program had started (April to May 2021), and the third survey (T3) was distributed after the summer when most COVID-19–related restrictions had been lifted (September to October 2021). Figure 1 shows the timeline and the severity of the COVID-19 pandemic in the Netherlands by means of excess mortality rates. Data were derived from Statistics Netherlands [34].

### Study Population and Recruitment

The surveys were disseminated via organizations working with people with MID or low literacy skills, such as advocacy organizations, care facilities for people with MID, language education organizations and libraries supporting and educating people with no or low literacy skills, social workplaces, the Dutch center of expertise on health disparities Pharos, Special Olympics, and a website offering accessible web-based information and programs for people with limited digital or literacy skills [35]. The surveys were open for between 4 and 6 weeks, giving the organizations time to distribute them within their network. Support was available to allow people who were anxious or unable to complete the survey independently to participate in this study. At the end of each survey, participants were asked to participate in future research, resulting in a panel of participants who could be contacted directly for the subsequent survey rounds.

Our easy-read survey was also distributed within the same time period to 2 municipal health service (MHS) panels: GGD Gelderland-Zuid (n=approximately 2500) and GGD Gelderland-Midden (n=approximately 7000). Each MHS panel consisted of residents in its service area who are regularly asked to complete health surveys. There is a known bias to these panels, in that they generally consist of older and more highly educated residents, with an overrepresentation of women [36]. We particularly used these panels in our study to obtain comparative data from a Dutch population sample and because these panels were also invited to participate in the national RIVM survey.

All participants received the same survey. Participation was voluntary, and participants could stop completing the survey at any time. Completion of a prior survey was not mandatory for participation in the next round. Data were obtained anonymously; therefore, matching between surveys and paired within-group analysis over time were not possible.

### Web-Based Survey Development

The RIVM survey on Dutch citizens’ perception of the COVID-19-related measures, their impact on well-being, and whether people were complying formed the basis of this monitor but was adapted to provide an easy-read version for this study [6]. The national survey consisted of >100 questions (eg, about participants’ well-being, trust in the government, adherence to COVID-19–related measures, the risk of COVID-19 infection, and the understanding of COVID-19 information). Over time, new topics such as willingness to receive COVID-19 vaccinations and vaccine hesitancy were added to the survey. To create an easy-read version, we adapted the national survey in three steps by (1) shortening the survey (ie, we selected only a limited number of relevant topics), (2) reducing the number of response categories, and (3) adjusting the language level. This procedure was based on literature insights [37] and carried out in collaboration with professionals (researchers: n=2, care providers: n=3, and policy makers: n=2 working with people with MID or low literacy skills regarding health-related issues) as well as 2 experiential experts with MID or low literacy skills trained to advise, and experienced in advising, research projects. First, to shorten the survey, we discussed, prioritized, and selected the most relevant topics in light of our research objective to measure mental health, relevance to the target population, and the target population’s cognitive capacity to answer the questions. Second, we reduced the number of response categories, in terms of Likert-scale options [38,39], by verifying the distinctiveness between the response categories and checking the relevance of the categories [40]. During the third and final step, abstract concepts, time references, and the language level were adjusted [41].

The easy-read survey consisted of 40 to 60 questions, with the length depending on the answers given to previous questions. The survey was pilot-tested with people with MID or low literacy skills (n=6) using the think-aloud methodology in cognitive interviews [42]. We used this method to verify the intended constructions of the questions and to assess the language level and the fit of the questions and response categories. Next, a web-based version of the survey was created on a web-based platform (called *I Coresearch*) designed with and for people with MID [43]. The platform has a clear layout, the possibility to enlarge font size, icons that can be added to response categories, and a speech-to-text and text-to-speech function. Additional pilot tests were carried out in which we observed participants (n=4-7 in each wave) while they were completing the survey to evaluate and improve the usability of the platform. The observation sessions were followed by retrospective interviews. The tests resulted in minor adjustments to the questions, the response categories, and the web-based platform. This procedure resulted in a final short easy-read web-based survey.

These same steps were followed to modify and revise the survey for the second and third rounds. After each survey round, the findings were discussed in 4 to 5 focus groups with either people with MID or low literacy skills or care and support professionals and policy makers concerned with these subgroups. This not only led to a quick dissemination of our findings accompanied by solutions or practical tips to put into practice, but the group discussions also provided input for the subsequent survey rounds in which questions that became less relevant over time (eg, adherence to specific measures and difficulty coping with changes in specific daily activities) were replaced by new questions (eg, about vaccination).

### Measures

The easy-read survey consisted of various topics, and we report the measures used for this specific study only (for details, refer to Multimedia Appendix 1).

#### Demographics and Contextual Factors

Similar to the national survey, an extensive section on demographics was included, such as age, gender, educational level, country of birth, and living situation. Furthermore, contextual factors known to have a potential influence on mental well-being were selected from the national survey and included, such as health status (eg, a rating of physical health and whether the participant had experienced COVID-19 infection), having social contacts, and socioeconomic status (eg, work status and the cessation of main activities because of COVID-19–related restrictions) [24,25]. To fit our target group’s everyday experience, *work status* included paid work, volunteer work, school, and day care. In addition to the national survey questions, we included *receiving professional care* because this is an important characteristic describing the support needs of our target population, as well as *survey completion methods* (alone or with help), about which participants with MID or low literacy skills were asked (refer to Multimedia Appendix 1 for all questions listed in the easy-read questionnaire).

#### Mental Health

To gain a better understanding of the impact of the COVID-19 pandemic on mental health, a final set of 6 outcome measures regarding mental health were defined. The RIVM survey incorporated a mix of newly developed and existing validated scales or items in its well-being module, including psychological well-being (the 5-item Mental Health Inventory [44]), loneliness (the 6-item De Jong Gierveld Loneliness Scale [45]), life satisfaction, resilience, positive and negative effects experienced due to the COVID-19 pandemic, and emotional response (ie, the extent of worry, stress, or fear people experience) to monitor various aspects of mental health during the COVID-19 pandemic [6]. The most relevant items for our target group and research aim were selected from this set in the first developmental step of item generation. Subsequently, the response format was evaluated. For reasons of uniformity, the response categories in this series of questions were all adapted to a 4-point Likert scale. All questions had to be revised in accordance with the language level and understanding of the target group, and we ensured that overlapping concepts were avoided. These steps resulted in the following questions: (1) “Did you feel happy in the last couple of days?” (2) “Did you feel full of energy in the last couple of days?” (3) “'Did you worry in the last couple of days?” (4) “Did you feel stressed in the last couple of days?” (5) “Did you feel lonely in the last couple of days?” and (6) “Did you have problems falling asleep in the last couple of days?” The outcomes were measured on a 4-point Likert scale: 1=*yes, almost always*; 2=*yes, often*; 3=*yes, sometimes*; and 4=*no, almost never* (Multimedia Appendix 1).

### Statistical Analyses

Mental health was measured at 3 time points over a 1-year period among people with MID or low literacy skills (referred to as the target panel) as well as among members of the MHS panels. First, we calculated the frequencies and medians of the descriptive and contextual measures at each time point for the target panel and the MHS panels. To assess differences between the panels, in each round, Pearson chi-square tests were conducted for nominal or ordinal variables, and nonparametric *t* tests (2-tailed) were performed for age. Second, we calculated the differences in frequencies of mental health scores using Pearson chi-square tests between survey rounds within each panel and between panels for each survey round. Third, we analyzed the impact of the group differences on mental health using linear regression analyses, while controlling for gender- and age-related differences. Given the large number of participants in each round and the multitude of comparisons made in analysis, differences and associations were considered statistically significant if *P* values were <.01 [46]. Statistical analyses were conducted in SPSS (version 25.0; IBM Corp).

### Ethical Considerations

The study was reviewed by the medical research ethics committee of Radboud University Medical Center, which ruled that this study did not fall under the Medical Research Involving Human Subjects Act and was therefore exempt from formal ethical review (2020-7033). We conducted the study in accordance with the General Data Protection Regulation and standard operating procedures of our research center.

All participants received the survey after they had been fully informed, in plain language, about the purpose of this study. All participants provided web-based written informed consent regarding participation and the use of their data for this study and for future purposes before filling out each questionnaire. For each survey, 20 vouchers worth €50 (US $53.9) each were raffled among people with MID or low literacy skills as motivation for participation.

Contact information used for the purpose of this raffle or future research was obtained and saved in a separate environment so that survey data could be obtained anonymously. Therefore, matching between surveys and paired within-group analysis over time were not possible.

## Results

### Participant Characteristics

Our web-based survey and adapted recruitment procedure enabled 1059 persons with MID or low literacy skills to participate (T1: n=412, 38.9%; T2: n=351, 33.1%; and T3: n=296, 28%). Background and contextual characteristics per survey round for the target panel and the MHS panels are presented in Table 1. Over the 3 time periods, 46.6% (138/296) to 53.2% (219/412) of the participants with MID or low literacy skills were women, with median ages ranging from 42 (IQR at T1: 27-57; IQR at T3: 28-54) to 45 (IQR 30-57 at T2) years and >70% (T1: 292/412, 70.9%; T2: 253/351, 72.1% and T3: 221/296, 74.7%) reporting no education or a low educational level. The majority (299/412, 72.6% at T1; 295/351, 84% at T2 and to 253/296, 85.5% at T3) were born in the Netherlands, 49.5% (204/412) to 62.2% (184/296) received professional care, and 22.6% (93/412) to 31.8% (94/296) reported living in a residential setting. Approximately half of the respondents (217/412, 52.7% at T1; 164/351, 46.7% at T2 and 170/296, 57.4% at T3) in each round reported very good or good physical health.

A total of 9305 MHS panel members completed our survey (T1: n=2930, 31.49%; T2: n=3213, 34.53%; and T3: n=3162, 33.98%). On the MHS panels over the 3 survey rounds, 55.85% (1766/3162) to 62.53% (1832/2930) of the participants were women, with median ages ranging from 52 to 62 years, and 12.73% (373/2930 at T1) to 17.08% (540/3162 at T3) had no education or a low educational level (ie, >70% had an intermediate or advanced educational level). The majority (2790/2930, 95.22% at T1; 3073/3213, 95.64% at T2 and 3023/3162, 95.6% at T3) were born in the Netherlands, only 3.28% (96/2930 at T1; 92/3213, 2.86% at T2 and 105/3162, 3.32% at T3) received professional care, and <1% (6/2930, 0.2% at T1; 6/3213, 0.19% at T2 and 3/3162, 0.1% at T3) lived in a residential setting. In addition, 77.12% (2478/3213 at T2 and 2435/3162, 77% at T3) to 79.39% (2326/2930 at T1) reported having very good or good physical health.

Altogether, this suggests that we successfully included a sample that represented our target population (ie, people with MID or low literacy skills). In addition, the characteristics of the MHS panels resemble those of the national sample, which is often used to represent the general Dutch population [9,36].

**Table 1:** Distribution of demographic and contextual characteristics (health and socioeconomic status, social contacts, and target group–specific characteristics) per survey round by target panel and municipal health service (MHS) panels^a^.

Characteristics		T1^b^ target panel (n=412)	T1 MHS panels (n=2930)	T2^c^ target panel (n=351)	T2 MHS panels (n=3213)	T3^d^ target panel (n=296)	T3 MHS panels (n=3162)
Age (y), median (IQR)		*42 (27-57)^e^*	*52 (41-64)*	*45 (30-57)^e^*	*60 (45-71)*	*42 (28-54)^e^*	*62 (46-72)*
Gender^f^ (woman), n (%)		*219 (53.2)^g^*	*1832 (62.5)*	185 (52.7)^h^	1865 (58)	*138 (46.6)^e^*	*1766 (55.9)*
**Educational level^e^, n (%)**							
	No to primary education	*91 (22.1)*	*12 (0.4)*	*63 (17.9)*	*17 (0.5)*	*82 (27.7)*	*25 (0.8)*
	Low	*201 (48.8)*	*361 (12.3)*	*190 (54.1)*	*477 (14.8)*	*139 (47)*	*515 (16.3)*
	Intermediate	*53 (12.9)*	*769 (26.2)*	*64 (18.2)*	*811 (25.2)*	*45 (15.2)*	*790 (25)*
	Advanced	*27 (6.6)*	*1730 (59)*	*8 (2.3)*	*1848 (57.5)*	*12 (4.1)*	*1785 (56.5)*
	Other^i^	*39 (9.5)*	*56 (1.9)*	*25 (7.1)*	*59 (1.8)*	*17 (5.7)*	*46 (1.5)*
Born in the Netherlands, n (%)		*299 (72.6)^e^*	*2790 (95.2)*	*295 (84)^e^*	*3.073 (95.6)*	*253 (85.5)^e^*	*3.023 (95.6)*
**Living situation^e^, n (%)**							
	Alone	*101 (24.5)*	*388 (13.2)*	*123 (35)*	*495 (15.4)*	*88 (29.7)*	*516 (16.3)*
	With family	*209 (50.7)*	*2525 (86.2)*	*149 (42.5)*	*2708 (84.4)*	*112 (37.8)*	*2636 (83.4)*
	In residential setting	*93 (22.6)*	*6 (0.2)*	*79 (22.5)*	*6 (0.2)*	*94 (31.8)*	*3 (0.1)*
Very good or good physical health, n (%)		*217 (52.7)^e^*	*2326 (79.4)*	*164 (46.7)^e^*	*2478 (77.1)*	*170 (57.4)^e^*	*2435 (77)*
COVID-19 infection, n (%)^j^		—	—	61 (17.4)^k^	461 (14.3)	45 (15.2)^l^	364 (14.7)
**Daily activities^m^, n (%)**							
	Paid work	*145 (35.2)^e^*	*1.920 (65.5)*	*133 (37.9)^e^*	*1.628 (50.7)*	*117 (39.5)^n^*	*1.528 (48.3)*
	Volunteer work	92 (22.3)^o^	748 (25.5)	74 (21.1)^p^	652 (20.3)	62 (20.9)^q^	738 (23.3)
	School	*100 (24.3)^e^*	*154 (5.3)*	*42 (12)^e^*	*87 (2.7)*	*47 (15.9)^e^*	*73 (2.3)*
	Day care	*121 (29.4)^e^*	*43 (1.5)*	*103 (29.3)^e^*	*32 (1)*	*103 (34.8)^e^*	*30 (0.9)*
	Other (sport, hobby, care, other)	197 (47.8)^r^	1558 (53.2)	179 (51)^s^	1760 (54.8)	176 (59.5)^t^	1668 (52.8)
	None	32 (7.8)^u^	351 (12)	*19 (5.4)^e^*	*379 (11.8)*	*9 (3)^e^*	*373 (11.8)*
**Change in daily activity^v^, n (%)**							
	Reduced or stopped	*125 (30.3)^e^*	*385 (13.1)*	*98 (27.9)^e^*	*538 (16.7)*	*67 (22.6)^e^*	*229 (7.2)*
	Nothing to do (bored)^j^	—	—	*18 (5.1)^e^*	*54 (1.7)*	*19 (6.4)^e^*	*49 (1.5)*
**Social contacts, n (%)**							
	No one I can talk to	*42 (10.2)^e^*	*188 (6.4)*	*45 (12.8)^e^*	*134 (4.2)*	20 (6.8)^w^	135 (4.3)
	No one who can help me	*59 (14.3)^e^*	*103 (3.5)*	*34 (9.7)^e^*	*88 (2.7)*	*24 (8.1)^e^*	*79 (2.5)*
Receives professional care, n (%)		*204 (49.5)^e^*	*96 (3.3)*	*216 (61.5)^e^*	*92 (2.9)*	*184 (62.2)^e^*	*105 (3.3)*
**Survey completion, n (%)**							
	Alone	187 (45.4)	—	202 (57.5)	—	169 (57.1)	—
	With help	223 (54.2)	—	147 (41.9)	—	127 (42.9)	—

^a^Category totals do not always add up to 100% because some categories (I don’t know and I don’t want to answer) and item nonresponse are not shown. Percentages are based on presented variable totals per category.

^b^T1: first survey (November to December 2020).

^c^T2: second survey (April to May 2021).

^d^T3: third survey (September to October 2021).

^e^Value for the target panel is significantly different from that for the regional panel (*P*<.001). Italicized values emphasize significance.

^f^<1% indicated their gender as “other.”

^g^Value for the target panel is significantly different from that for the regional panel (*P*=.001). Italicized values emphasize significance.

^h^Value for the target panel is not significantly different from that for the regional panel (*P*=.15).

^i^The answer category “other” is chosen by respondents when it does not fit any of the provided options. This may be because they do not know their educational level, do not recognize their education category from the option list, or they were educated in a country other than the Netherlands.

^j^This question was added to the survey from T2.

^k^Value for the target panel is not significantly different from that for the regional panel (*P*=.12).

^l^Value for the target panel is not significantly different from that for the regional panel (*P*=.72).

^m^Respondents could provide multiple answers; the category total can therefore add up to than 100%.

^n^Value for the target panel is significantly different from that for the regional panel (*P*=.005). Italicized values emphasize significance.

^o^Value for the target panel is not significantly different from that for the regional panel (*P*=.16).

^p^Value for the target panel is not significantly different from that for the regional panel (*P*=.73).

^q^Value for the target panel is not significantly different from that for the regional panel (*P*=.38).

^r^Value for the target panel is not significantly different from that for the regional panel (*P*=.04).

^s^Value for the target panel is not significantly different from that for the regional panel (*P*=.18).

^t^Value for the target panel is not significantly different from that of the regional panel (*P*=.03).

^u^Value for the target panel is not significantly different from that for the regional panel (*P*=.01).

^v^This variable is constructed concerning the daily activities of paid work, volunteer work, and day care.

^w^Value for the target panel is not significantly different from that for the regional panel (*P*=.02).

### Mental Health

**Figure 2:**
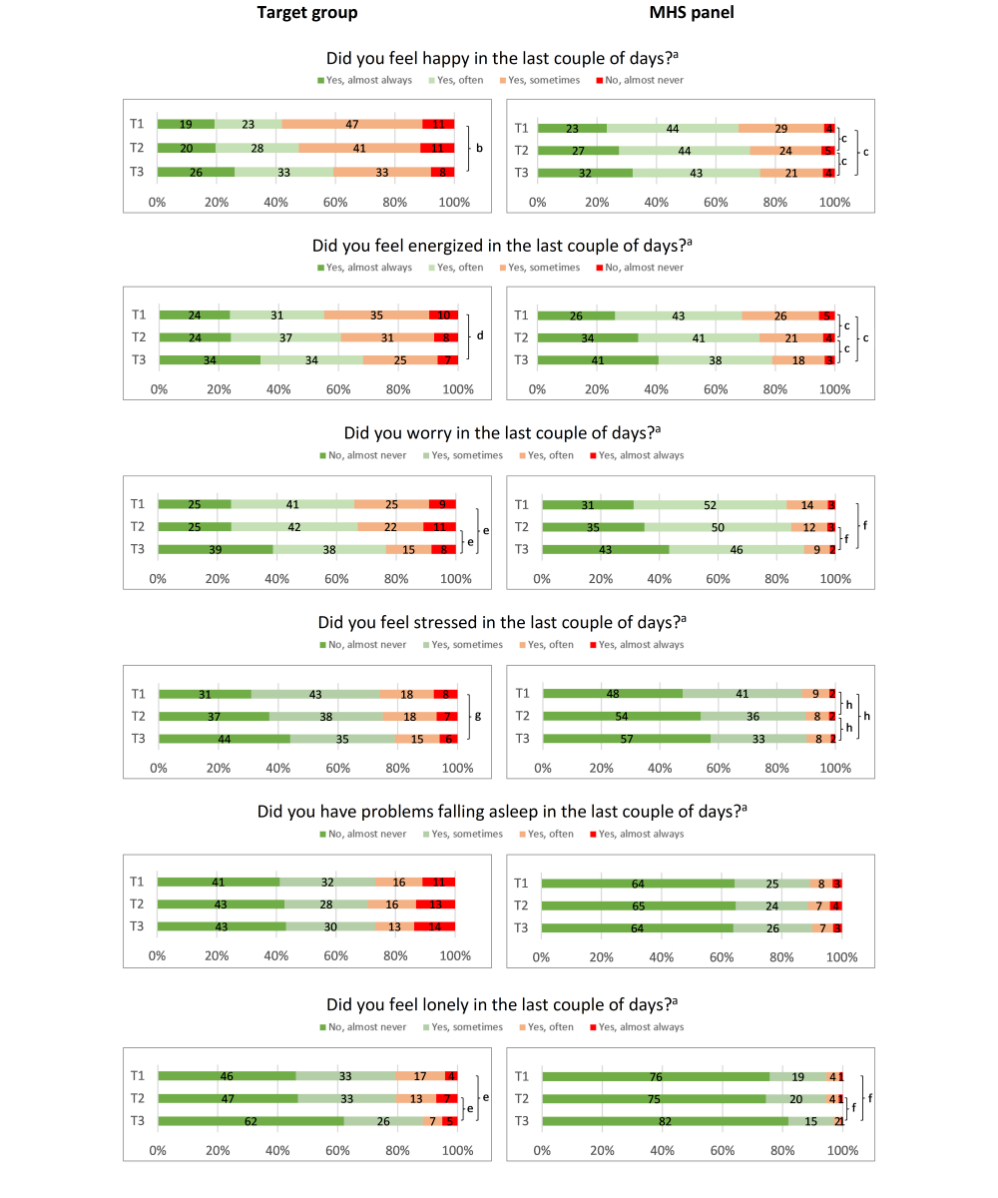
Distribution of mental health outcomes for participants in the target panels and members of the municipal health service (MHS) panels at 3 different time periods. T1: first survey; T2: second survey; T3: third survey. ^a^Difference between groups within each round (*P*<.001), ^b^Difference between rounds within target group (T1 vs T3: *P*<.001), ^c^Difference between rounds within panel (T1 vs T2, T2 vs T3, and T1 vs T3: *P*<.001), ^d^Difference between rounds within target group (T1 vs T3: *P*=.002), ^e^Difference between rounds within target group (T2 vs T3, and T1 vs T3: *P*<.001), ^f^Difference between rounds within panel (T2 vs T3, and T1 vs T3: *P*<.001), ^g^Difference between rounds within target group (T1 vs T3 *P*=.005), ^h^Difference between rounds within panel (T1 vs T2, and T1 vs T3: *P*<.001 and T2 vs T3 *P*=.01).

The analyses of the distributions of the frequencies of mental health scores within the target panel show no differences between T1 and T2. There are significant differences between T1 and T3 regarding feeling happy, feeling energized, feeling stressed, worry, feeling lonely, and sleeping problems among people with MID or low literacy skills (Figure 2) and between T2 and T3 for these aspects, except for worry and feeling lonely; the percentage of people reporting positive feelings *often* or *almost always* increased, and the percentage of people reporting negative feelings *often* or *almost always* decreased over time. There were no differences observed regarding sleeping problems.

Regarding the MHS panels, there were significant differences between each survey round for feeling happy, feeling energized, and feeling stressed. For worry and feeling lonely, significant differences were observed only between T3 and the 2 previous rounds. The direction of the differences is similar to that observed in the target panels. Similar to the target panels, the MHS panels did not report differences regarding sleeping problems.

The analyses between the different panels within the survey rounds show that the percentage of participants in the target panel reporting negative feelings on mental health outcomes was significantly higher compared with the members of the MHS panels, especially within T1 and T2 (Figure 2); for example, looking at the 6 outcome measures within T1, the percentages of people who reported more negative feelings range from 20.8% (85/408) feeling lonely *often* or *almost always* to 58.3% (238/408) feeling happy *almost never* or only *sometimes*. The MHS panels show a different and more positive distribution on all outcome measures. The percentages of people on the MHS panels who reported more negative feelings range from 5.5% (160/2904) feeling lonely *often* or *almost always* to 32.2% (941/2918) feeling happy *almost never* or only *sometimes*. Figure 2 presents more details on the distribution of all mental health outcomes for the target panel and the MHS panels.

### Impact of Literacy Skills on Mental Health

Regression analyses adjusted for age and gender by using them as covariates show that the differences found between the 2 panels exist for almost all mental health outcomes in each survey round, except feeling stressed in T3 (*P*=.07). In addition, the differences between the panels during T1 (age- and gender-adjusted β ranging from −0.376 to 0.525) are larger than those observed in T3 (age- and gender-adjusted β ranging from −0.257 to 0.509), except for sleeping problems. Table 2 presents details for all time periods and outcome measures.

**Table 2:** Results for each survey round with panel as independent variable, mental health measures as dependent variables, and gender and age as covariates.

		T1^a^			T2^b^			T3^c^	
		β (95% CI)	*P* value	β (95% CI)		*P* value	β (95% CI)		*P* value
**Feeling happy**									
	Panel	−*0.386*^d^ *(−0.464 to −0.287)*	*<.001*	−*0.385 (−0.481 to −0.288)*		*<.001*	−*0.257 (−0.362 to −0.152)*		*<.001*
	Gender	−0.037 (−0.096 to 0.022)	.22	−0.092 (−0.151 to −0.034)		.002	−0.065 (−0.122 to −0.07)		.03
	Age	0.000 (−0.002 to 0.002)	.75	0.000 (−0.002 to 0.002)		.86	0.000 (−0.002 to 0.002)		.77
**Feeling energized**									
	Panel	−*0.156 (*−*0.249 to* −*0.063)*	*.001*	−*0.243 (*−*0.340 to* −*0.145 )*		*<.001*	−*0.143 (*−*0.249 to −0.037)*		*.008*
	Gender	−0.039 (−0.102 to −0.023)	.22	−0.080 (−0.139 to −0.021)		.008	−0.058 (−0.117 to 0.000)		.05
	Age	0.004 (0.002 to 0.006)	<.001	0.003 (0.001 to 0.005)		.003	0.004 (0.002 to 0.006)		<.001
**Worry**									
	Panel	*0.328 (0.246 to 0.409)*	*<.001*	*0.324 (0.255 to 0.429)*		*<.001*	*0.227 (0.135 to 0.319)*		*<.001*
	Gender	0.158 (0.104 to 0.213)	<.001	0.217 (0.165 to 0.270)		<.001	0.181 (0.131 to 0.232)		<.001
	Age	0.000 (−0.001 to 0.002)	.60	−0.002 (−0.004 to 0.000)		.02	−0.002 (−0.003 to 0.000)		.06
**Feeling stressed**									
	Panel	*0.274 (0.195 to 0.353)*	*<.001*	*0.199 (0.117 to 0.281)*		*<.001*	0.081 (−0.168 to 0.006)		.07
	Gender	0.158 (0.105 to 0.211)	<.001	0.189 (0.140 to 0.239)		<.001	0.195 (0.147 to 0.243)		<.001
	Age	−0.010 (−0.012 to −0.008)	<.001	−0.012 (−0.014 to −0.011)		<.001	−0.013 (−0.014 to −0.011)		<.001
**Sleeping problems**									
	Panel	*0.525 (0.439 to 0.611)*	*<.001*	*0.518 (0.427 to 0.609)*		*<.001*	*0.509 (0.411 to 0.606)*		*<.001*
	Gender	0.220 (0.163 to 0.278)	<.001	0.296 (0.241 to 0.351)		<.001	0.240 (0.187 to 0.294)		<.001
	Age	0.003 (0.001 to 0.005)	.002	0.000 (−0.002 to 0.002)		.90	0.002 (0.000 to 0.003)		.09
**Feeling lonely**									
	Panel	*0.479 (0.409 to 0.549)*	*<.001*	*0.461 (0.387 to 0.535)*		*<.001*	*0.297 (0.229 to 0.365)*		*<.001*
	Gender	0.095 (0.049 to 0.142)	<.001	0.161 (0.116 to 0.206)		<.001	0.071 (0.034 to 0.109)		<.001
	Age	−0.001 (−0.003 to 0.000)	.10	−0.002 (−0.004 to −0.001)		.002	−0.002 (−0.003 to 0.000)		.01

^a^T1: first survey (November to December 2020).

^b^T2: second survey (April to May 2021).

^c^T3: third survey (September to October 2021).

^d^Italicized values indicate significant regression results, with *P*<.01 level.

## Discussion

### Principal Findings

This is the first study to monitor the mental health and well-being of people with MID or low literacy skills and a general population sample over the course of 1 year during the COVID-19 pandemic. With our adapted web-based survey co-designed with representatives from our target population, we were able to reach subgroups that are usually underrepresented in surveys. Our study showed that feelings of happiness, energy, worry, stress, and loneliness improved in both populations over the course of the pandemic. However, the COVID-19 pandemic and related restrictions had a much bigger impact on the mental health of people with MID or low literacy skills than on that of the general population.

In general, our findings show that, during the second lockdown in the Netherlands (ie, at the time of the first survey round), people with MID or low literacy skills as well as the general population sample reported poorer mental well-being than 1 year later when all restrictions were lifted, and the COVID-19 infections became less severe (ie, at the time of the third survey round). These findings are in line with research on people in vulnerable positions [1] as well as the general population who experienced fewer negative feelings over the course of the COVID-19 pandemic [5]. Previous literature has shown that the impact on mental health and well-being is correlated to the stringency of disease control measures [25,47]; for example, the closure of social care services, workplaces, and day care activities negatively influenced daily structure and social interactions [29-31,48,49], thereby increasing stress and anxiety [50], and quarantines and social isolation were found to have an effect on loneliness, fear, and boredom [27,28,51]. When these measures were relaxed, there was a partial improvement in mental health [47]. Although our study was not designed to prove any causation between the stringency of disease control measures and mental health impact, our findings show a similar pattern of decreasing worries, stress, and loneliness whereas feelings of happiness and energy increased over time. Notably, this was also the case in our general population sample [1,3,5,14,25,26]; however, the pandemic disproportionately impacted people with MID or low literacy skills, who reported more negative mental health outcomes in all survey rounds.

Qualitative studies among people with MID show that long-term social restrictions in particular had an extensive impact on their daily life by limiting social connections and work activities [27,29]. Our target population reported these limitations in daytime activities to a greater extent than the general population sample. Interviews by Voermans et al [29] provide more in-depth assessment of the consequences of these limitations for people with MID, showing a major impact in terms of social isolation, difficulties coping with negative thoughts, struggles with autonomy in society, stigmatization, a lack of routine and purpose, boredom, and lower self-worth. As awareness is raised about the significant value of meaningful social contacts and daytime activities, professionals and policy makers should provide tailored policies that consider both health risks and the risks of social isolation. Societal participation initiatives should be organized and sustained for people with MID or low literacy skills, both during and outside of a pandemic.

Besides the disruptive impact of disease control measures on the target population’s daily routines and social contacts, the high levels of confusion and uncertainty that resulted from the rapidly changing measures as well as fear and loss of control may have played a role in their reduced mental health [32] in periods of both stringent measures and relaxation of control measures [14]. In addition, people with lower health literacy skills are known to have less resilience, which affects their feelings of anxiety, stress, or worry [52,53], thereby putting them at greater risk of mental health problems. Ongoing support should be provided to enhance resources of resilience and coping strategies in people with MID or low literacy skills through either formal or informal caregivers.

Our findings highlight the need to prioritize the mental health consequences of the pandemic and the disease control measures for people with MID or low literacy skills [1,54,55]. The majority of our sample received support from formal and informal caregivers, who are an important source of support. Studies have shown detrimental effects on the mental health of these caregivers as well [28,56]. Therefore, we suggest tailoring generic disease control measures to the specific situations of groups considered vulnerable and their support system, instead of widely implementing measures such as social distancing, visiting restrictions, and closure of schools or day care facilities (eg, by developing strategies to maintain social inclusion during pandemic challenges through a combination of supportive carers, assisted digital communication technologies, and safe social activities) [57,58]. Hence, engaging groups considered vulnerable and their support system in policy making and decision-making is essential in the tailoring process [59].

This study underscores the relevance of including people with MID or low literacy skills in health research and therefore endorses current calls to action in practice and science [60]. Health information systems are crucial for providing data for policy making and decision-making, but the underrepresentation of people with MID or low literacy skills in health data may lead to biased policy decisions, with adverse and detrimental effects on existing health disparities [13,33]. Previous research has suggested that, to reduce disparities and guide policy, researchers should evaluate how health outcomes are distributed among specific demographic groups and compare these distributions with those of the overall population [61], as was done in our study. Collecting information about people with MID or low literacy skills should become routine in demographic and public health data collection. We have shown that, by co-designing an adapted survey and using an accessible web-based platform and specific recruitment procedures, it is possible to collect information among people with MID or low literacy skills, even during lockdown periods; for example, the sample characteristics showed that participants with MID or low literacy skills differed from the general population in educational level, country of birth, and daily activities. With our adjusted approach to data collection, we were quickly able to obtain relevant information about people with MID or low literacy skills and disseminate our findings and recommendations, thereby facilitating policy makers to guide disease control measures and health promotion activities that address the immediate as well as longer-term health needs of people with MID or low literacy skills or other populations considered vulnerable.

### Limitations of the Study

Executing a repeated cross-sectional survey among people with MID or low literacy skills during a pandemic is fraught with challenges. Therefore, our study has some limitations. First, we did not collect longitudinal data because we wanted to lower the threshold for participation by choosing an anonymous design. In addition, people were not obliged to complete all 3 surveys. As a result, it was impossible to track individual participants over time. Second, inevitably, the validated questions had to be revised to include people with MID or low literacy skills in our survey. However, we tested the questions in cognitive interviews, and the project team worked as inclusively as possible together with the target population to create a valid survey to obtain reliable data. More than half of the participants over all survey rounds (558/1059, 52.69%) were able to complete the web-based survey themselves, and support was arranged for the remaining group of respondents (497/1059, 46.93%). This should encourage future researchers to consider easy-read web-based surveys among people with MID or low literacy skills as long as target group representatives are closely involved in designing and testing these surveys. Third, our study started after the onset of the COVID-19 pandemic and lacks a baseline measurement of mental health before the pandemic. Therefore, it remains inconclusive as to whether people with MID or low literacy skills experienced greater mental health problems during the COVID-19 pandemic than before the pandemic. Fourth, we relied mostly on organizations in our network (eg, health care organizations and public libraries with special literacy programs) to contact and recruit people with MID or low literacy skills. Besides the possible sampling bias that this may have caused, we could not track how many people were approached to take part in the survey. Therefore, we were unable to report information about response rates. Fifth, because the survey was conducted on the web, those without access to the internet or sufficient digital literacy skills may have been excluded. Sixth and last, there was also a bias in our general population sample. However, although the sample was not representative of the Dutch general population in terms of age, gender, and educational level, it allowed us to contextualize our findings and gain a deeper understanding of the challenges faced by people with MID or low literacy skills compared with the general population sample, while controlling for differences in gender and age. We were able to do so because we used the same easy-read questionnaire in both groups to prevent survey bias. An open-ended question about the experience of members of the MHS panels with this type of questionnaire at T3 revealed that the majority of participants (1829/2337, 78.26%) appreciated this approach, given that a broader population was enabled to participate. Although this may indicate that easy-read questionnaires can be used for broader purposes and other populations, rather than being aimed specifically at people with low literacy skills alone, this single question does not provide sufficient information regarding a broad survey approach, and more research is required.

### Conclusions

In conclusion, our study enabled insight into the impact of the COVID-19 pandemic and related control measures on the mental health of people with MID or low literacy skills. General disease control measures for the entire Dutch population had a more negative impact on people with MID or low literacy skills than on the general population. Although mental health improved over the course of the pandemic in both populations as the COVID-19–related restrictions were gradually lifted, and the severity of the disease decreased over time, the disproportional effect remained. Professionals should be aware of this and pay attention to the needs of people with MID or low literacy skills in research, practice, and policy by tailoring measures that consider physical, social, and mental health effects and providing support to overcome such effects.

This study underscores the relevance of including people with MID or low literacy skills in public health research because they are often overlooked in regular health data. An accessible and structural web-based monitor for people with MID or low literacy skills enabled us to do so and provides better knowledge for care providers and policy makers to react to unexpected events such as a pandemic. To prevent existing health disparities from increasing, greater account should be taken of the impact of control measures on people who are relatively more vulnerable.

## Appendix

Multimedia Appendix 1 Overview of all questions of the easy-read survey included in this study and response categories provided, separated into demographics and contextual measures, and in mental health items.

## Abbreviations

ID: intellectual disability
MHS: municipal health service
MID: mild intellectual disability
RIVM: Rijksinstituut voor Volksgezondheid en Milieu (National Institute for Public Health and the Environment)

## Data Availability

The data sets generated and analyzed during this study are available in the Radboud Data Repository.
