# Supplementary material for: An Accessible Web-Based Survey to Monitor the Mental Health of People With Mild Intellectual Disability or Low Literacy Skills During the COVID-19 Pandemic: Comparative Data Analysis
Source: JMIR Public Health Surveill. 2024 May 30;10:e44827. doi: 10.2196/44827 (PMC11176870; doi:10.2196/44827)
Supplement: Multimedia Appendix 1 [file publichealth_v10i1e44827_app1.docx]

**MULTIMEDIA APPENDIX 1:**

**Overview of all questions of the easy-read survey included in this study and response categories provided, separated into demographics and contextual measures, and in mental health items.**

| Demographics and contextual factors | Question | Answer categories provided | Adjusted for descriptive analyses |
| --- | --- | --- | --- |
| Demographics |  |  |  |
| Age | How old are you? | Fill in, years |  |
| Gender | Are you a man or a woman? | 1=Man; 2=Women; 3=Other |  |
| Educational level | What the last school that you attended? | 1=None; 2=Basic education; 3=Special primary education; 4=Secondary special education; 5=Lower vocational education; 6=Pre-vocational secondary education; 7=Higher general secondary education; 8=Vocational education; 9=Higher education or university; 96=Other | 1 OR 2 OR 3=None or basic; 4 OR 5 OR 6=Low; 7 OR 8=Middle; 9=High;  Other=96 |
| Born in the Netherlands | Are you born in the Netherlands? | 0=Yes; 1=No |  |
| Living situation | Who do you live with? | 1=I live alone; 2=I live with, for example, my partner, children, parents or friends; 3=I live in a residential setting |  |
| Health status |  |  |  |
| Physical health | What is your physical health like? | 1=Very bad; 2=Bad; 3=Sometimes bad, sometimes good; 4=Good; 5=Very good |  |
| COVID-19 infection | Have you had a COVID-19 infection | 1=Yes, I tested positive; 2=Yes, I think so but I did not perform a test; 3=No, I don’t think so | 1=Yes (1)  2 OR 3=No (0) |
| Socioeconomic status |  |  |  |
| Daily activities | What daily activities did you do before the COVID-19 pandemic?  *With multiple answers:* What did you do the most before the COVID-19 pandemic? | 1=Paid work; 2=Volunteer work; 3=School; 4=Providing informal care; 5=Daycare; 6=Sport; 7=Hobby; 8=Other; 9=None | 4 OR 6 OR 7 OR 8=Other  Answers of this question are reported |
| Changes in daily activities due to  COVID-19 | Has anything changed in your <*most important daily activity*> since the COVID-19 pandemic? | 1=Yes, my activity was stopped; 2=Yes, my activity has changed; 3= Yes, my activity is reduced; 4= Yes, my activity is increased; 5= Yes, my activity is now remotely from home; 6=No, nothing is changes | 1 OR 3=Yes (1) |
| Boredom | How often did you have nothing to do during the day over the past few days? | 1=almost always 2=I often had nothing to do  3=Sometimes I had nothing to do; 4=hardly ever | 1 OR 2=Nothing to do |
| Social contacts |  |  |  |
| Presence of social contacts - trust | Do you have people you can tell everything to? | 1=Yes; 2=No; 3=I don’t want to tell anything to other people | 1 OR 3=Yes (0)  2=No (1) |
| Presence of social contacts - help | Do you know people who can help you if you need help? | 1=Yes; 2=No; 3=I don’t need any help | 1 OR 3=Yes (0)  2=No (1) |
| Characteristics of target population in study |  |  |  |
| Receiving professional care | Do you receive help at home such as care or support? | 1=Yes, from a professional; 2=Yes, from family or friends, 3=No | 1=Yes  2 OR 3 = No |
| Survey completion | Are you completing the questionnaire alone or with another person? | 1=Alone; 2=With help from someone I know; 3=With help from someone I didn’t know before the survey | 1=Alone  2 OR 3=With help |

| Mental health*^1^* | Question | Answer categories provided | Adjusted for descriptive analyses |
| --- | --- | --- | --- |
| Happiness | Did you feel happy the past few days? | 1=Yes, Almost always; 2=Yes, Often; 3=Yes, Sometimes; 4=No, Almost never |  |
| Feeling energized | Have you felt like doing things in the past few days? | 1=Yes, Almost always; 2=Yes, Often; 3=Yes, Sometimes; 4=No, Almost never |  |
| Worry | Have you been worried the past few days? | 1=Yes, Almost always; 2=Yes, Often; 3=Yes, Sometimes; 4=No, Almost never | 1=4; 2=3; 3=2; 4=1  Scale reversed for presentation purposes |
| Feeling stressed | Have you felt stressed the past few days? | 1=Yes, Almost always; 2=Yes, Often; 3=Yes, Sometimes; 4=No, Almost never | 1=4; 2=3; 3=2; 4=1  Scale reversed for presentation purposes |
| Sleeping problems | Did you find it difficult to fall asleep the past few days? | 1=Yes, Almost always; 2=Yes, Often; 3=Yes, Sometimes; 4=No, Almost never | 1=4; 2=3; 3=2; 4=1  Scale reversed for presentation purposes |
| Feeling lonely | Have you felt lonely the past few days? | 1=Yes, Almost always; 2=Yes, Often; 3=Yes, Sometimes; 4=No, Almost never | 1=4; 2=3; 3=2; 4=1  Scale reversed for presentation purposes |
| ^1.^ All questions provided with an explanation regarding the time indication, was stated ‘Think of yesterday, the day before yesterday and the days before that.’ | | | |
